# The effect of human amniotic epithelial cells on urethral stricture fibroblasts

**Published:** 2019-07-21

**Authors:** Sanjay Gottipamula, Sudarson Sundarrajan, Kumar Chokalingam, K. N. Sridhar

**Affiliations:** ^1^Sri Research for Tissue Engineering Pvt. Ltd., Shankara Research Centre, Bengaluru, Karnataka, India; ^2^Cancyte Technologies Pvt. Ltd., Rangadore Memorial Hospital, Bengaluru, Karnataka, India

**Keywords:** Alpha-smooth muscle actin, Amniotic epithelial cells, Fibrosis, Tissue inhibitor of metalloproteinases-1, Urethral stricture

## Abstract

**Background:**

Urethral stricture disease (USD) is effectively managed by buccal mucosa (BM) urethroplasty. Lack of adequate healthy BM has led to the use of autologous tissue-engineered BM grafts. Such grafts are costly, not easily scalable and recurrence of the stricture is still a problem. Hence, there is a requirement for cost-effective, scalable cells with innate antifibrotic properties which seem to be fulfilled by human amniotic epithelial cells (HAMECs). The effect of HAMECs on USD is unknown.

**Aim:**

To study the effect of HAMECs-CM on human urethral stricture fibroblast (USF) cells by using *in-vitro* migration assay and molecular techniques.

**Materials and Methods:**

USF cells were derived from six patients undergoing urethroplasty. HAMECs were derived from one placenta after delivery. The effect of HAMECs-CM on USF cell migration was observed using a standard *in vitro* scratch assay over a period of 3 days. The effect of HAMECs-CM on the expression levels of markers alpha-smooth muscle actin (α-SMA) and tissue inhibitor of metalloproteinases (TIMP-1) in USF cells was also examined.

**Results:**

The HAMECs-CM suppressed the migration of USF cells in *in vitro* scratch assay. The HAMECs-CM consistently downregulated α-SMA, but not TIMP-1.

**Conclusions:**

HAMECs have shown antifibrotic activity on USF cells in this *in vitro* study.

**Relevance for Patients:**

HAMECs could serve as an alternative cell source for tissue-engineered urethroplasty.

## 1. Introduction

Urethral stricture disease (USD) is a condition that leads to urethral narrowing due to fibrosis. The fibrosis may be in response to the damage of urethra either due to infection, inflammation, or trauma [1,2]. Progressive fibrosis leads to obstruction of the urethra, thereby causing infection, sepsis, calculi, and renal failure [3]. The estimated prevalence rate of USD is approximately 1% of population with prevalence mostly in older men [3]. The estimated cost of treating USD exceeded US $190 million in 2000 [4].

The management of USD consists of dilatation, primary anastomosis, stents, urethroplasty using grafts, and flaps. Among urethroplasty procedures, buccal mucosa (BM) graft urethroplasty is very popular as the success rate is high [5,6]. In situations, where BM is either fibrosed or diseased, substitution urethroplasty might not be possible. Hence, tissue-engineered BM (TEBM) using cultured autologous BM cells seeded onto scaffolds has been developed and demonstrated to be safe and effective [7-10]. However, the study by Bhargava *et al*. showed that fibrosis and contraction occurred in two of the five patients within 1 year [7]. MukoCell® as a product had an overall success rate of 67.3% at 12 months and 58.2% at 24 months and in other study found the success rate was 80-85.7% at 55 months [8,9]. Our experience with autologous TEBM grafts revealed narrowing at the anastomotic region [10].

These studies suggest that recurrence of stricture is still a problem with TEBM urethroplasty. Biopsy of BM for TEBM generation has its own morbidity, depends on the condition of BM, and it is not scalable or cost effective. Hence, a new source of cells that can be generated in large numbers with minimal effort, cost, and exhibiting profound antifibrotic effects is advantageous. The epithelial cells from amniotic membrane (human amniotic epithelial cells [HAMECs]) have been explored for their non-immunogenic, antifibrotic [11], anti-inflammatory [12], and genetic stability [11]. This has led to the generation of allogeneic scalable and cost-effective modules for various clinical applications [13,14].

Alpha-smooth muscle actin (α-SMA) and tissue inhibitor of metalloproteinases (TIMP-1) have been shown to induce fibrosis by their upregulation at molecular level in various fibrotic conditions including urethral stricture [15-19]. It is hypothesized that antifibrotic therapy should downregulate these markers and also decrease the proliferation of fibroblast [20], which may result in decrease of fibrosis.

The epithelial cells may act through paracrine factors [21-23] which can be extracted in conditioned media (CM). This CM can be used to study the antifibrotic activity of HAMECs cells. The effect of HAMECs on USD has not been explored in humans.

Hence, in this study, we evaluated the effect of HAMECs-CM on urethral stricture fibroblast (USF) cells using *in vitro* scratch assay and gene expression of α-SMA and TIMP-1.

## 2. Materials and Methods

### 2.1 Isolation and cell culture

The collection of tissues was approved by Rangadore Memorial Hospital Institutional Ethics Committee and informed consents were obtained from all donors.

Biopsies of urethral scar tissues from male USD patients undergoing urethroplasty (*n*=6, 42-72 years) were transported to the cell isolation facility in a cold medium containing Hank’s Balanced Salt Solution (Gibco, USA) and 1% antibiotic-antimycotic (Gibco, USA). The USF cells were isolated and expanded as per our previously published protocol [24]. The USF cells isolated by digesting stricture tissues in Collagenase Type IV (Life Technologies, USA) were cultured in Dulbecco’s Modified Eagle Medium (DMEM) (Life Technologies, USA) supplemented with 10% fetal bovine serum (FBS) (Hyclone, USA) in standard conditions till passage 2 (P2).

Placenta (*n*=1, 32 years) from healthy mother who underwent elective cesarean was transported to the cell isolation facility. The HAMECs were isolated and expanded as per our earlier published protocol [11]. Briefly, cut pieces of amnion were digested in 0.25% Trypsin (Gibco, USA) at 37°C in an orbital shaker for multiple rounds of 40 min each to isolate HAMECs. The HAMECs were cultured in KnockOut (KO) DMEM (Life Technologies, USA) supplemented with 10% FBS, 2 mM Glutamax (Life Technologies, USA), 1% antibiotic-antimycotic, and 10 ng/ml epidermal growth factor (Sigma, USA) (KO DMEM complete media) under standard conditions until P1.

The morphology of USF cells at P2 and HAMECs at P1 was imaged with an inverted microscope (Nikon TE 200, Tokyo, Japan).

### 2.2 CM collection

The HAMECs cells were cultured in CellStacks One (Corning, USA) at P1 and media change was done at 50-60% confluency using freshly prepared KO DMEM complete media. CM was collected at time when HAMECs were at 80-90% confluency and filtered using 0.2-µm syringe filter (Millex-GP syringe filter, Millipore, Ireland) and stored at −20°C until further use. Before use, the collected HAMECs-CM was thawed and diluted with equal amount of basal DMEM media (CM). Fresh complete DMEM media diluted to 50% with basal DMEM media henceforth referred to as FM, served as controls.

### 2.3 In vitro scratch assay

An *in vitro* scratch assay was performed as per our previously published protocol [20]. There were modifications which included USF cells at P2 seeded at 5000-7000 cells per cm^2^ in 6-well plates (CellStar, Greiner Bio-One, Germany) until 90-100% confluency. The USF cells were incubated overnight in basal DMEM media containing 0.5% FBS. A scratch was made on the confluent layer of cells using a 1000 ml pipet tip (Greiner Bio-One, Germany). Cells were then gently washed with basal DMEM media and then incubated with 2 ml of either CM or FM. The cell migration into the scratch area was monitored and imaged for 3 days. Experiments were done in triplicates for six batches of USF cells. The images were analyzed using the wound healing tool macro in Image J software (version 1.48V, National Institutes of Health, Bethesda, MD).

### 2.4 Gene expression studies

The 0.5×10^5^ USF cells at P2 were seeded in 6-well plates (CellStar, Greiner Bio-One, Germany) and cultured in DMEM containing 10% FBS until they reached 80-90% confluency. The cells were incubated overnight with DMEM containing 0.5% FBS and then incubated for 24 h in either FM or CM. About 1 ml of (Life Technologies, USA) was added to the wells after Dulbecco’s phosphate-buffered saline (Gibco, USA) wash. Total RNA was extracted from respective USF cells using TRIzol Plus RNA purification kit (Invitrogen, USA) according to the manufacturer’s instructions. The RNA concentration was measured using NanoDrop (Thermo Scientific, USA). Total RNA was equalized in all samples and subjected to HL-ds DNase (ArcticZymes, Norway) treatment to remove genomic DNAs and then transcribed to cDNA using SuperScript III Platinum one-step quantitative reverse transcription-polymerase chain reaction (PCR) kit (Invitrogen, USA) as previously described [24]. About 2 µl of the prepared cDNA was subjected to quantitative PCR (qPCR) using Maxima SYBR Green qPCR master mix to determine the expression of α-SMA, TIMP-1, and glyceraldehyde-3-phosphate dehydrogenase (GAPDH).

The conditions for qPCR were initial denaturation 95°C for 5 min, followed by 40 cycles of 95°C for 20 s, 58°C for 30 s, and 72°C for 30 s. At the end of the PCR cycles, the melting curve was analyzed for specific amplified product. The gene expression of α-SMA and TIMP-1 was normalized to GAPDH to check the relative gene expression. The experiments were performed in six batches of USF cells in triplicates.

### 2.5 Statistical analysis

The normalized relative gene expression levels and the percentage of gap values were expressed as mean ± standard error of the mean and the data were analyzed by t-test using GraphPad Prism (Version 5, GraphPad Software, USA). *P*≤0.05 was considered statistically significant.

## 3. Results

### 3.1 Cell morphology

**Figure 1:**
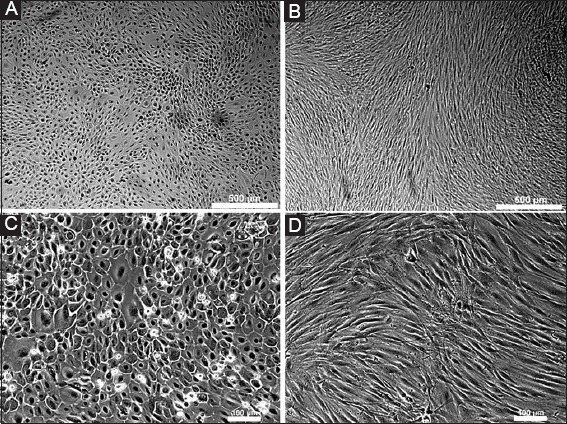
Morphology of human amniotic epithelial cells at passage 1 (P1) **(A, C)** and urethral stricture fibroblast cells at P2 **(B, D)**. The magnification of top row is ×4 **(A, B)** and the bottom row is ×10 **(C, D)**. Scale bar: 500 µm (×4) and 100 µm (×10).

Morphology of the USF cells and HAMECs was visualized and imaged using an inverted microscope. The USF cells at P2 were uniformly spindle shaped with tapering ends (Figure 1B and D) as previously described [24]. The HAMEC cells at P1 were cuboidal, innate characteristics of epithelial cells (Figure 1A and C) as previously described [11,25,26].

### 3.2 In vitro scratch assay

**Figure 2:**
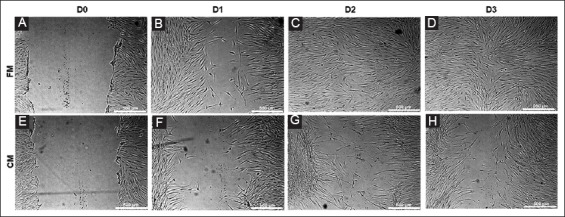
**(A-H)** Representative images of *in vitro* scratch assay on urethral stricture fibroblast cells. Top row represents fresh media and bottom row represents conditioned media and column represents time points. Scale bar: 500 µm.

**Figure 3:**
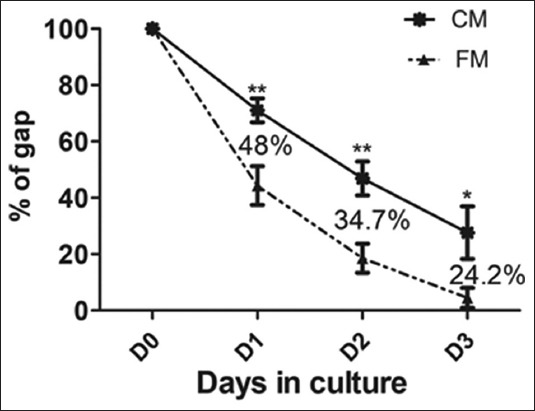
Percentage of gap on day 0 (D0), day 1 (D1), day 2 (D2), and day 3 (D3) in FM and conditioned media (CM). The numbers in the figure represent the differential percentage of gap between FM and CM at the respective time points. **P<0.01: *P<0.05.

To evaluate the effect of HAMECs-CM on migration of USF cells, an in vitro scratch assay model was used and observed over a period of 3 days. The CM reduced the migration of USF cells compared to FM (Figure 2 and 3). On day 0, the scratch area created, was devoid of any cells (Figure 2A and E). The gap closure of USF cells in CM was significantly slow on all days compared to FM (Figure 3) and the difference of reduction of gap by FM and CM on days 1-3 was 48%, 34.7%, and 24.2%, respectively. However, we did see a variation in degree of migration between different donors.

### 3.3 Gene expression of α-SMA and TIMP-1

Gene expression levels for α-SMA and TIMP-1 were performed to evaluate whether HAMECs-CM downregulated these genes in USF cells compared to FM.

**Figure 4:**
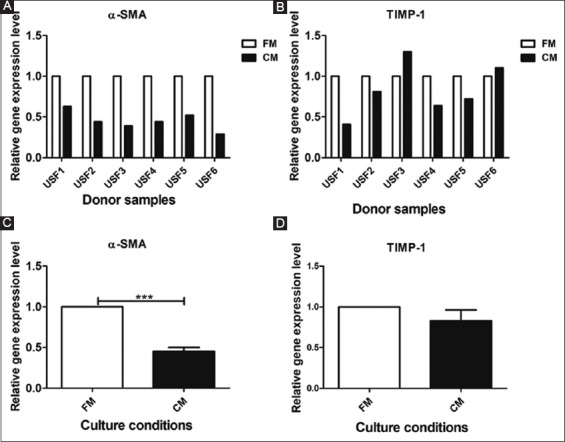
Relative gene expression of alpha-smooth muscle actin and tissue inhibitor of metalloproteinases-1 in urethral stricture fibroblast cells cultured in FM and conditioned media. Top row **(A, B)** shows individual donor samples and bottom row **(C, D)** shows the average relative expression. ***P<0.001.

The CM-treated USF cells showed a minimum of 37% downregulation of α-SMA (range 37%–71%) in all the samples compared to FM as shown in Figure 4A. Overall, the reduction was 55% and this reduction was significant (*P*<0.05) (Figure 4C).

The gene expression of TIMP-1 showed downregulation in four donor USF cells (19%–59%) (Figure 4B). Overall, CM-treated USF cells showed 17% reduction in TIMP-1 expression, which was not significant (Figure 4D).

## 4. Discussion

The USD is a challenge to urologists as recurrent fibrosis is still a problem following treatment [27]. Although BM graft is a significant progress in the surgical treatment of USD, it is associated with donor site morbidity and unavailability of consistent healthy tissue for reconstruction. These limitations of BM grafts have led to the emergence of autologous cell-seeded tissue-engineered grafts. Autologous tissue-engineered grafts are not easily scalable, costly, and recurrence of the stricture is still a problem [10]. Accordingly, there is a need to explore cost-effective [28,29], scalable cells with innate antifibrotic properties. HAMECs are one such cell source which seem to fulfill the above requirements [14,30-32].

HAMECs and their secretome, which are present in the CM, have been shown to abrogate fibrosis [14,21,30-32]. However, the effect of HAMECs on USD is unknown. Hence, we investigated the suitability of HAMECs in reducing fibrosis of USD, by studying the effects of HAMECs-CM on the expression of α-SMA and TIMP-1, in USF cells and their migration under *in vitro* conditions.

We evaluated the expression of biomarkers α-SMA and TIMP-1 in USF as these have prominent roles in urethral fibrosis. The α-SMA had high expression levels in urethral fibrous tissue [15,16] and correlated with increased collagen production [15]. The importance of α-SMA in fibrosis is highlighted in an animal model where lung fibrosis was attenuated by inhibition of α-SMA [17]. Urethral stricture had higher levels of TIMP-1 expression and correlated with increased collagen levels [15]. Upregulation of TIMP-1 also induced an increase in stricture fibroblasts growth and invasion [15]. The suppression of TIMP-1 reduced the urethral scar formation [18] by decreased secretion of collagen [19] in an animal model.

In our study, the HAMECs-CM significantly reduced α-SMA in USF cells. Similar observation of downregulation of α-SMA in keloid fibroblast by HAMECs-CM [32] and the alleviation of fibrosis in an animal model [17,31] allow us to speculate that downregulating α-SMA in USD may lead to reduction in fibrosis. Hence, HAMECs with their ability to downregulate α-SMA could be a potential antifibrotic therapy for urethral fibrosis.

Furthermore, TIMP-1 was not consistently downregulated by HAMECs-CM. The variation observed in the TIMP-1 expression in our study (Figure 4) might be attributed to its pleiotropic activity [33] or the intrinsic variation of strictures [34] from different donors. TIMP-1 may, therefore, not be a reliable marker for antifibrotic assay.

Other than antifibrotic and anti-inflammatory properties of HAMECs, the fact that these cells can be easily obtained in large numbers from discarded placental tissue with less ethical concern supports the use of these cells as a potential therapy in USD. With regards to bringing this technology to practice, the HAMECs would be isolated from placentae, screened for infections and critical genetic mutations, and then grown onto scaffolds that would be grafted subsequently grafted into patient’s urethra. The isolation, expansion, and characterization and genetic stability of HAMECs were published previously [11]. The average yield of isolated HAMECs is around 100 million cells from a single placenta and these can be expanded to P1 with a yield of approximately 700 million cells, which is sufficient to create 150 urethral grafts. The HAMECs have been cultured up to P4 without any impact on genotype and phenotype [35]. The HAMEC batches would undergo standard quality testing as per internal specifications. The cells from these batches would be loaded on scaffolds and cultured. This would be the investigational medicinal product for transplantation. It should be noted that the HAMECs morphological characteristics, identity (AE1/AE3), and ability to attach and grow in culture (monolayer) were similar to that of a scaffold environment (unpublished data). Corroboratively, Rashedi *et al*. have demonstrated that the paracrine effects in cell monolayers and scaffolds were similar, for example, the downregulation of α-SMA [36]. However, efficacy in preclinical animal models of USD and humans has to be demonstrated as per regulatory guidelines before clinical use.

The main significance of our study is the use of USF cells that are directly taken from patient stricture region and considering that these USF cells might be critical factor in the development of fibrosis. Conventionally, the antifibrotic potency assays have been conducted using cell lines [37,38].

This study has limitations in being an *in vitro* study, CM has not been characterized and USF cells probably vary with strictures. The testing on biopsy tissue would be more representative than enriched cells. On the other hand, the enriched cells would provide us reproducible environment to test various parameters. This study has been limited to the paracrine effects of HAMECS at P1 monolayer instead of cell-seeded scaffolds, which is the final product. At present, we have not studied the cryogenic storage of HAMECs or CM across different stability time point to check the fibrosis-ameliorating effects (potency assay) of the cells. Finally, there are no available data in literature to show the variability of secretome composition in HAMECs with increasing population number. This could be taken up in the long-term stability program of potency assay.

## 5. Conclusions

HAMECs-CM significantly reduced α-SMA expression and migration of USF cells demonstrating its antifibrotic properties. Hence, HAMECs could be an alternative cell source for the generation of cost-effective TE replacement grafts for USD. Further, *in vivo* studies are needed to evaluate the antifibrotic effect of HAMECs. To the best of our knowledge, this is the first study to evaluate the effects of HAMECs-CM on human USF cells.
